# Structural determinants of the scorpion venom peptide Uy234 govern bactericidal activity and membrane-disruptive properties

**DOI:** 10.3389/fmicb.2026.1830314

**Published:** 2026-06-11

**Authors:** Ana Karen Villa-Merlan, Andrea Mescola, Pedro Alejandro Fong-Coronado, Víctor Rivelino Juárez González, Flora Fernández-Sánchez, Andrea Alessandrini, Daniel Balleza, Verónica Quintero-Hernández

**Affiliations:** 1 Laboratorio de Biología Molecular y Biotecnología, Centro de Investigaciones en Ciencias Microbiológicas, Instituto de Ciencias, Benemérita Universidad Autónoma de Puebla, Puebla, Mexico; 2 CNR-Nanoscience Institute-S3, Modena, Italy; 3 Dirección de Posgrado en Ciencias en Biotecnología, Universidad Politécnica del Estado de Morelos, Jiutepec, Mexico; 4 Departamento de Medicina Molecular y Bioprocesos, Instituto de Biotecnología, Universidad Nacional Autónoma de México, Cuernavaca, Mexico; 5 Laboratorio de Microbiología, Unidad de Investigación y Desarrollo en Alimentos (UNIDA), Instituto Tecnológico de Veracruz, Tecnológico Nacional de México, Veracruz, Mexico; 6 Department of Physics, Informatics and Mathematics, University of Modena and Reggio Emilia, Modena, Italy; 7 Secretaría de Ciencia, Humanidades, Tecnología e Innovación (SECIHTI), Mexico City, Mexico

**Keywords:** antimicrobial peptide, bacterial cell viability, lateral expansion, membrane permeability, peptide flexibility, Uy234 peptide

## Abstract

**Introduction:**

The growth-inhibiting effect of the peptide Uy234, present in the venom of the scorpion *Urodacus yaschenkoi*, has been investigated in two bacterial pathogens: *Staphylococcus aureus* ATCC 25923 and *Acinetobacter baumannii* AE12, the latter being a multidrug-resistant clinical isolate. With the aim of determining the possible role of specific residues in the bioactivity of this peptide, we studied a proline residue at position 9 and the C-terminal amidation of this peptide.

**Methods:**

Two inactivated variants were analyzed: Uy234-C, a non-amidated peptide, and Uy234-A, a P9A mutant. In addition to quantifying in detail the minimum inhibitory and bactericidal concentrations for each microorganism, membrane-damaging effects were assessed through bacterial cell viability assays with SYTO9/PI fluorophores. In addition, AFM, electroforming, and GUV microaspiration were used to determine the effects of each peptide in terms of permeabilization. Molecular dynamics (MD) simulations were also performed for the wild-type peptide and its P9A mutant.

**Results:**

Only the native peptide Uy234 showed bacteriostatic and bactericidal activity, whereas the P9A mutant and non-amidated variant lost antimicrobial activity, demonstrating the essential role of the Pro-9 residue and C-terminal amidation in Uy234 bioactivity against both pathogens. SYTO9/PI assays in *S. aureus* infection showed membrane damage only with native Uy234, while AFM and GUV studies revealed membrane thinning, lateral expansion, and dose-dependent permeabilization of lipid bilayers.

**Discussion:**

Our study provides clear evidence of a damaging effect on the membrane associated with the bioactivity of Uy234. This bioactivity is directly associated with the presence of residue P9 and the presence of C-terminal carboxyamidation. The mutant peptide P9A is unable to permeabilize GUVs, which is consistent with the persistence of a greater degree of structural order, according to MD simulations in the aqueous phase. This study provides a framework for the rational design of bactericidal peptides targeting multidrug-resistant bacteria.

## 1 Introduction

The rapid emergence of bacterial resistance to conventional antibiotics represents a major global health challenge (Murray et al., 2022). One promising strategy involves exploring natural sources of antimicrobial peptides, including animal exudates and the venoms of reptiles and arthropods. The venoms of many scorpions, for example, are a natural source of a wide variety of bioactive components with a broad range of pharmacological activities. These peptides are divided into two groups: (1) cysteine-rich peptides with disulfide bridges and (2) cysteine-free peptides without disulfide bonds (Almaaytah and Albalas, 2014; Fong-Coronado et al., 2024). Cysteine-rich peptides include neurotoxins that act on different types of ion channels and are responsible for the poisoning symptoms observed in humans after scorpion stings (Quintero-Hernández et al., 2013). On the other hand, cysteine-free peptides have attracted considerable attention due to their valuable biological activities, including cytolytic, anticancer, immunomodulatory, and bradykinin-potentiating activities, and, most importantly, significant antimicrobial activity (Ortiz et al., 2015; Fong-Coronado et al., 2024). Remarkably, many of these peptides exhibit potent antimicrobial activity, inhibiting the growth of *ESKAPE* pathogens (Pedron et al., 2019; Oliveira et al., 2021; Mechkarska et al., 2023). The venom of *Urodacus yaschenkoi*, the Australian scorpion, is composed of a cocktail of proteolytic and bioactive agents with bactericidal potential. One of those components is a short antimicrobial peptide (ssAMP) described as Uy234, an 18-residue peptide exhibiting a +3 (+1/+2) net charge (Luna-Ramírez et al., 2013, 2015). Uy234 adopts a partial amphipathic α-helical structure in solution, as determined by circular dichroism experiments (Cesa-Luna et al., 2019). Furthermore, Uy234 exhibits a strongly hydrophobic character, in addition to the presence of a proline residue at position 9, which confers it significant flexural mobility. This intrinsic flexibility is associated with its C-terminal amidation and an intramolecular network of hydrogen bonds that stabilize the peptide in α-helical configurations. These physicochemical attributes facilitate the internalization of the peptide into the lipid bilayers, thereby killing several *ESKAPE* bacteria, probably by promoting micellization phenomena (Salazar-Hernández et al., 2024). In particular, this ssAMP has been evaluated against various clinical isolates, including pathogens such as *Acinetobacter baumannii* ATCC 17978, *Klebsiella pneumoniae*, *Streptococcus* spp., and some methicillin-resistant (MRSA) *Staphylococcus aureus* strains (Cesa-Luna et al., 2019). This peptide has attracted considerable attention due to its low hemolytic activity, which supports its therapeutic potential (Cesa-Luna et al., 2019; Salazar-Hernández et al., 2024).

Despite this important information, the actual mechanisms governing the bioactive behavior of the Uy234 peptide are still unknown, and therefore remain as a subject of debate. Although it is believed that Uy234 acts on the lipid matrix of bacterial cell membranes, destroying their barrier properties and contributing to kill bacteria, it is still not quite clear whether this is the only mechanism involved. It is important to consider, for example, that unlike long peptides such as melittin (26-residues), which are capable of stabilizing toroidal pores in membranes (Naito et al., 2000; Leveritt et al., 2015), peptides as short as Uy234 are unlikely to fully span the ∼4 nm thickness of a lipid bilayer as single monomers. This fact has led to various proposals for the mechanisms of action of short AMPs, which include micellization and detergent effects (Balla et al., 2004; Li et al., 2006), pore formation by dimerization (Lorenzón et al., 2013; Balatti et al., 2020) or lipid segregation and an increase in lipid packing defects (Fernandez et al., 2012, 2013).

In this study, the interactions of the Uy234 peptide and two presumably inactivated forms of that peptide, i.e., a peptide mutated at position 9 by an alanine residue (Uy234-A) and the peptide in its carboxylated, non-amidated, version (Uy234-C), were investigated in detail. These three peptides were tested against *S. aureus* ATCC 25923 and *A. baumannii* AE12. Studies were also conducted with model membranes using supported lipid bilayers (SLBs) and atomic force microscopy (AFM), as well as permeability studies in Giant Unilamellar Vesicles (GUVs). Furthermore, a fluorescence assay was performed on *S. aureus* to directly evaluate damage to the membrane of living cells. Finally, we performed molecular dynamics simulations in aqueous phase for the Uy234-A mutant and compared the results with those of the active peptide, which includes a Pro residue at that position (Uy234). We show that Uy234 is capable of effectively killing *A. baumannii*; we also demonstrate that the bioactivity of this peptide against *S. aureus* strongly depends on the Pro9 residue, which appears to be a key structural factor for the lytic activity of Uy234. Similarly, we demonstrated that the interaction of Uy234 with lipid membranes that simulate the typical lipid composition found in bacteria induces thinning, fluidization, and lateral expansion effects. We propose that these mechanical effects are directly linked to the real-time permeabilization of liposomes, which we also report in this paper. Taken together, we provide key insights into the basic principles underlying the lytic activity of this ssAMP and its potential as a future therapeutic alternative against *ESKAPE* bacteria.

## 2 Materials and methods

### 2.1 Bacterial strains

All microbiological assays were performed in *Staphylococcus aureus* ATCC 25923 or Multidrug-resistant *Acinetobacter baumannii* AE12 (Supplementary Figure 1). Bacteria were cultured overnight in Luria–Bertani (LB) broth at 37 °C under constant orbital shaking (180 rpm). The overnight culture was subsequently diluted 1:100 in fresh LB medium and incubated under the same conditions until mid-logarithmic phase was reached, corresponding to an optical density at 600 nm (OD_600_) of approximately 0.5.

### 2.2 Peptides and lipids

Uy234 (FPFLLSLIPSAISAIKRL-NH_2_) and Uy234-A (FPFLLSLIASAISAIKRL-NH_2_), were synthesized by Abclonal Science, Inc. The non-amidated version of Uy234, i.e., Uy234-C (FPFLLSLIPSAISAIKRL-COOH) was synthesized by Peptide 2.0 Inc. Peptide purity was determined by analytical high-performance liquid chromatography (HPLC) according to the manufacturers’ reports. Following synthesis and purification, peptides were supplied in lyophilized form. For experimental evaluation, peptides were resuspended in a mixture of acetonitrile:distilled water (1:5, v/v). The physicochemical parameters of these peptides were estimated as previously reported (Salazar-Hernández et al., 2024).

Three kinds of lipids were also evaluated: 1-palmitoyl-2-oleoyl-sn-glycero-3-phosphoethanolamine (POPE), 1-palmitoyl-2-oleoyl-sn-glycero-3-phospho-(1’-rac-glycerol) (POPG), and 1,2-dioleoyl-sn-glycero-3-phosphocholine, or 18:1 (Δ9-Cis) PC (DOPC) were all purchased from Avanti Polar Lipids, Inc. (Alabaster, United States). Each lipid was dissolved in the proper amount of chloroform (SigmaAldrich Corp.) and then mixed with the other lipids of interest to create the desired lipid proportion. The lipid ratios in the reported mixtures represent molar ratios.

### 2.3 Evaluation of antimicrobial activity by radial diffusion

Antimicrobial activity was evaluated using a qualitative agar radial diffusion assay performed in triplicate with *S. aureus* or *A. baumannii*. For inoculum preparation, 500 μL of an overnight (O.N.) culture was transferred into 25 mL of fresh LB broth and incubated at 37 °C with shaking (180 rpm) until the culture reached a turbidity equivalent to the 0.5 McFarland standard (OD_625_ ≈ 0.08–0.12). Absorbance was measured using a UV–Vis spectrophotometer (METASH V-5100/UV-5100). The culture was then diluted 1:10 in fresh medium. A volume of 5 mL of this bacterial suspension was evenly spread onto LB agar plates using the lawn culture technique. After allowing adsorption for 1 min, excess inoculum was removed, and plates were dried for 30 min under a laminar flow hood with lids partially open to facilitate complete absorption. Once the inoculum had dried, 10 μL of each peptide (Uy234, Uy234-A, and Uy234-C) at a final concentration of 100 μM was deposited directly onto the agar surface. Vancomycin (50 μg/mL) was used as a positive control. Plates were incubated at 37 °C for 16 h, after which inhibition halos were evaluated.

### 2.4 Determination of the minimum inhibitory (MIC) and bactericidal (MBC) concentrations

The minimum inhibitory concentration (MIC) and minimum bactericidal concentration (MBC) were determined in triplicate by broth microdilution using sterile 96-well microplates, following the protocol described by Li et al. (2014) with minor modifications. In brief, bacteria were cultured O.N. in 5 mL LB broth at 37 °C with orbital shaking (180 rpm). Then, 500 μL of the O.N. culture was transferred into fresh LB medium and incubated under the same conditions until mid-log phase (OD_600_ ≈ 0.6). The culture was adjusted to approximately 5 × 10^5^ CFU/mL by dilution in fresh medium, based on a previously established correlation between OD_600_ and viable cell counts. Colony-forming units (CFU/mL) were counted in triplicate using the drop plate method, as described by Muñoz-Rojas et al. (2016). For MIC determination, two-fold serial dilutions (1:2) of the peptides were prepared in LB medium across a concentration range of 200, 100, 50, 25.5, 12.75, 6.3, 3.1, and 1.5 μM. Assays were conducted in ELISA microplates, including the following controls in triplicate: (1) diluent (acetonitrile:tetradistilled water), (2) sterile LB, (3) LB + bacteria, or (4) vancomycin as a positive control.

Absorbance was measured at 600 nm using a BioTek ELx800 microplate reader before and after 16 h incubation at 37 °C with shaking (180 rpm) (Li et al., 2014; Salazar-Hernández et al., 2024). The MIC was defined as the lowest peptide concentration at which no increase in absorbance relative to the initial value was observed, indicating inhibition of bacterial growth. For MBC determination, aliquots from the same microplate used for MIC assessment were transferred using a previously sterilized and irradiated metal replicator onto LB agar plates. Plates were incubated at 37 °C for 16–24 h. The MBC was defined as the lowest concentration at which no visible bacterial growth occurred on agar, corresponding to a ≥ 99.9% reduction in the initial population (Cesa-Luna et al., 2019). All assays were performed in triplicate.

### 2.5 Determination of membrane damage by SYTO9/PI fluorescence

Bacterial membrane integrity was assessed using the LIVE/DEAD BacLight kit™ (Thermo Fisher Scientific), based on differential staining with SYTO9 and propidium iodide (PI). Mid-log phase cells, obtained as previously described, were exposed to peptides (100 μM, 2 h), following the (Pazos-Rojas et al., 2019) protocol with modifications. Cells were subsequently stained and analyzed by fluorescence microscopy (VE-146YT, 100 × ) using filter B (420–490 nm) for SYTO9 and filter G (500–550 nm) for PI. Green fluorescence (SYTO9) was considered indicative of viable cells with intact membranes, whereas red fluorescence (PI) indicated non-viable cells with compromised membranes. Fluorescence was also quantified using an AMR-100 microplate reader, measuring SYTO9 emission at 492 nm and PI emission at 630 nm.

### 2.6 Supported lipid bilayers (SLB) and AFM

1-palmitoyl-2-oleoyl-sn-glycero-3-phosphoethanolamine (PO PE) and 1-palmitoyl-2-oleoyl-sn-glycero-3-phospho-(1’-rac-glycerol) (POPG) were purchased from Avanti Polar Lipids (Alabaster, United States) and used without further purification. Once the necessary amount of lipids in chloroform had been transferred to glass vials, the solvent was evaporated under a gentle flow of nitrogen and under vacuum to remove it completely. The lipid films formed on the glass were then rehydrated with buffer solution (150 mM KCl, 8 mM HEPES, 3 mM CaCl2, pH 7) and stirred until completely resuspended in the form of large multilamellar vesicles (LMVs). SUV were then obtained by reducing the size of the LMVs through sonication for 30 min at ∼40 °C. The SLBs were assembled on a freshly cleaved mica substrate in a homemade chamber equipped with a thermal control system mounted on the microscope stage. The vesicle fusion mechanism on mica allows for the formation of the planar bilayer. POPE:POPG (3:1 and 1:1) SLBs for AFM measurements were prepared by adding 100 μL of the SUV suspension (0.25 mg/mL) to 1.5 mL of buffer solution, and 70 μL of the SUV suspension was deposited onto the cleaved mica substrate. In both cases, the lipids were incubated for 15 min at ∼40 °C and then rinsed with buffer solution prior to analysis. The AFM experiments were performed as reported in our previous studies (Marín-Medina et al., 2018). Briefly, imaging was performed by a Bioscope (Veeco Metrology, CA, United States) equipped with a Nanoscope IIIa controller using the tapping-mode in liquid mode. The temperature of the sample was controlled by a water circulating bath below the sample and it was continuously controlled by a thermocouple inserted in the same imaging solution. The Uy234 peptide was injected in the imaging solution at a final concentration up to 12 μM.

### 2.7 GUV preparation and imaging

DOPC was purchased from Avanti Polar Lipids (Alabaster, United States) and used without further purification. Lipids were prepared by mixing solutions in chloroform until the desired amount (1 mg/mL). Bovine Serum Albumin (BSA) was purchased from Sigma Aldrich (Saint Louis, Missouri, United States). GUVs made of DOPC were grown using the electroformation method with minor modifications (Angelova and Dimitrov, 1986; Balleza et al., 2019). Basically, small drops (2–3 μL) of lipids were deposited on two opposing Pt wires (separation: 2–3 mm) inside a home-made Teflon chamber. Chloroform was removed by exposing those wires to a continuous N2 flux and then by using a vacuum pump (10–2 mBar) for at least 30 min. The circuit was connected to an electric potential wave generator set to produce a sinusoidal potential difference. The Teflon chamber was then filled with a 0.1 M sucrose solution and sealed using glass coverslips and vacuum grease (Dow-Corning, Midland, Michigan, United States). Electroformation protocol: (1) 10 Hz, 2.0–3.0 Vp-p for 1 h and 30 min; (2) 5 Hz, 2–2.5 Vp-p for 30 min, and (3) 2 Hz, 1.5 Vp-p for 15–20 min. The vesicle formation process was performed at ∼40°C. As a final step we applied a square wave at 5 Hz in order to promote GUV detachment from the wires. Before observation, glass slides were pretreated with BSA (10 mg/mL) to prevent surface adhesion effects on the sample. Thus, vesicle solution was diluted ∼10 times into a 105 mM glucose solution to create contrast asymmetry between the inside and the outside of the vesicles. Because of the differences in density and refractive index between both milieus, GUVs were deposited by gravity at the bottom of the observation chamber having better contrast when observed with phase contrast microscopy. Under these conditions, each peptide was added at a local concentration of 10 μM, while we monitored in real time the possible lytic effect of the Uy234 peptide and two variants thereof (Uy234-C, Uy234-A).

### 2.8 Molecular modeling and dynamic simulations

Uy234 and Uy234-A peptides were modeled with the AlphaFold3 (AF3) server. Both models were validated in terms of bond lengths, angle geometry, Ramachandran plots, and Z-score (Supplementary Figure 2). Molecular Dynamics (MD) simulations were carried out in triplicate using the GROMACS package (Abraham et al., 2015) via the WebGRO online resource.^1^ The parameters used for every system of protein in aqueous milieu include the GROMOS96 43a1 forcefield (FF), water model (SPC), box type (triclinic), salt concentration (NaCl 150 mM), energy minimization using an integrator steep descendent with 10,000 steps, and equilibration type NVT/NPT at 300 K, 1 bar, during 100 ns. The analysis of the results was expressed in terms of the Root Mean Square Deviation (RMSD) and Fluctuation (RMSF), the Radius of Gyration (Rg), and the time-averaged number of hydrogen bonds (H-bonding). The permeability assays of Uy234 and Uy234-A peptides in DOPC membranes at 300 K and pH 7 were performed using the Cell-PM software (Lomize and Pogozheva, 2018).

### 2.9 Statistical analysis

Results are reported as mean ± standard deviation (SD), calculated from independent experiments, and analyzed using SigmaPlot (Systat Software Inc.) for graphical representation and statistical evaluation.

## 3 Results

### 3.1 Physicochemical analysis of the peptides

**FIGURE 1:**
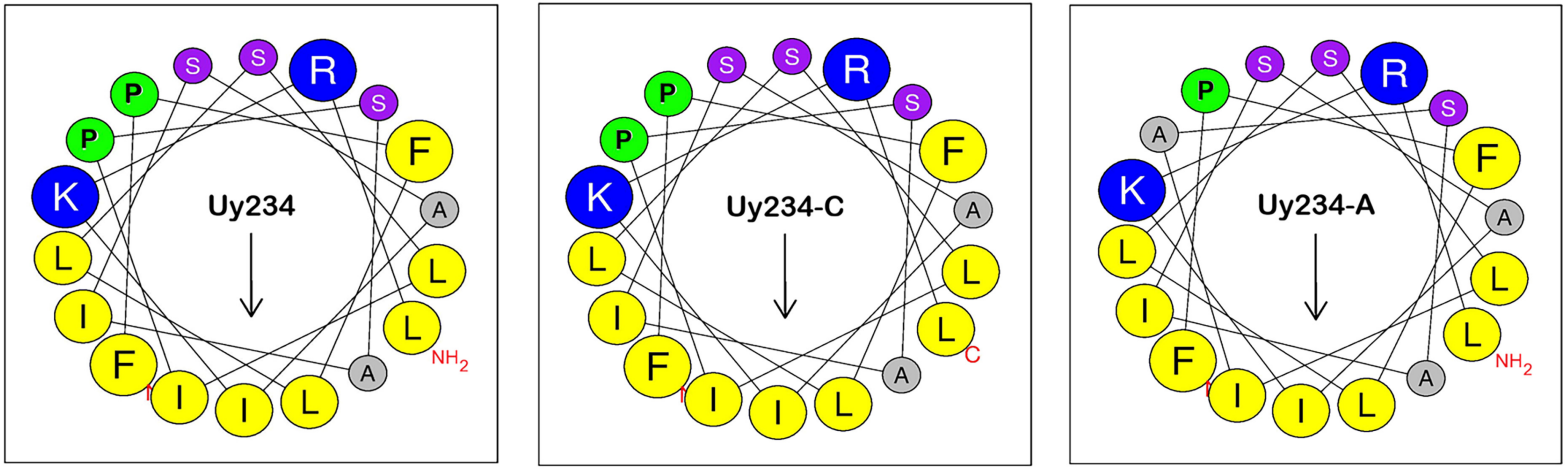
Helical wheel projections of Uy234 and two inactivated derivatives. Each residue is shown in proportion to the size of its side chain. Strongly hydrophobic residues are shown in yellow and those with basic or polar attributes, in blue and purple, respectively. Proline residues, which induce internal flexibility, appear in green; alanine, which strongly tends to stabilize α-helices, are depicted in grey. The hydrophobic vectors of the amide forms cannot be estimated in HeliQuest, so those data are approximate.

Some of the most relevant physicochemical properties of the three evaluated peptides are summarized in Table 1. The helical wheel projection of each peptide for each peptide is depicted in Figure 1, where the segregated distribution of hydrophobic and hydrophilic residues is clearly evident, highlighting their amphipathic characters. The spatial residue organization is consistent with the formation of an α-helical secondary structure, as it has been determined for the parental peptide, i.e., Uy234 (Cesa-Luna et al., 2019).

**TABLE 1:** Physicochemical parameters of Uy234, Uy234-C, and Uy234-A.

Peptide	Sequence	MW^a^ (Da)	NZC^b^	GRAVY^c^	H^d^	μH^e^	mBf ^f^	PIVA^g^	μ^h^ (Debye)	TEE ^i^ (ϕ) (kJ/mol)	Boman Index ^j^ (kJ/mol)
Uy234	FPFLLSLIPSAISAIKRL-NH**_2_**	1985.48	(+1/+2)	1.32	0.87	0.47	1.79	64.35	163	1.22 E4	**–**3.09
Uy234-C	FPFLLSLIPSAISAIKRL-COOH	1986.47	(0/+2)	1.32	0.87	0.47	1.79	67.35	163	1.21 E4	**–**3.09
Uy234-A	FPFLLSLI**A**SAISAIKRL-NH**_2_**	1959.44	(+1/+2)	1.51	0.85	0.48	1.67	701.45	178	1.27 E4	**–**3.51

^a^Mol. Weight in Daltons.

^b^Main and side chain charges, respectively.

^c^Grand Average hydropathy.

*^d^*Hydrophobicity according HeliQuest.

^e^Hydrophobic Moment according HeliQuest (Note: the μH of the amide forms cannot be estimated in Heliquest, so this data is approximate).

^f^Intrinsic flexibility in terms of the mean B-factor according to Balleza (2023).

^g^PIVA = Propensity to *in vitro* Aggregation according to DBAASP.

^h^Dipole moment according to Felder et al. (2007).

^i^TEE = Total Electrostatic Energy according APBS (Amber FF, pH 7).

^j^According APD6.

### 3.2 Evaluation of antimicrobial activity by the radial diffusion assay

**FIGURE 2:**
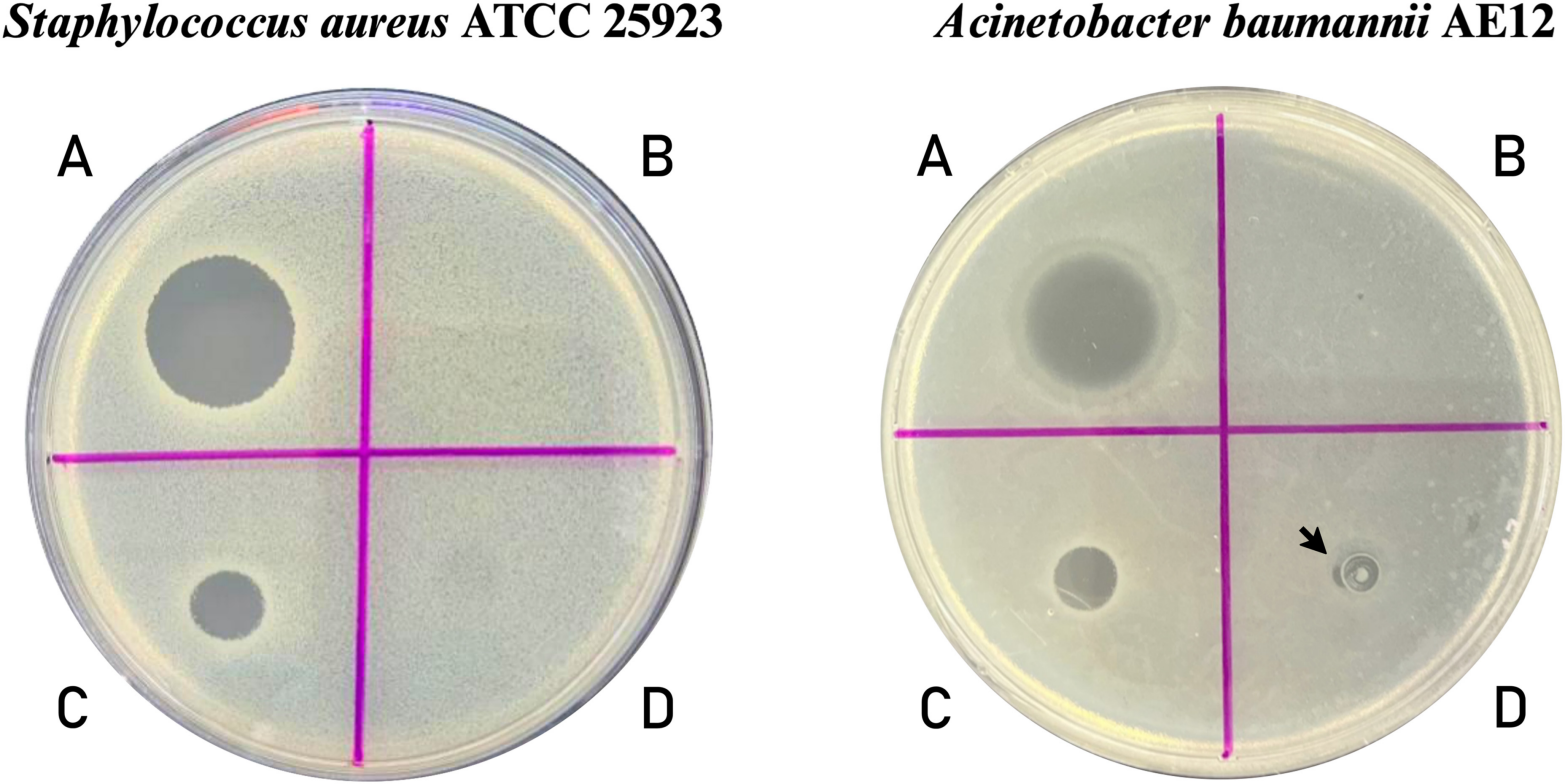
Antimicrobial activity of the three peptides determined by the radial diffusion assay. **(A)** Vancomycin (positive control). **(B)** Uy234-A. **(C)** Uy234. **(D)** Uy234-C. The formation of an inhibition halo indicates cell death and antimicrobial activity. The arrow shows a slight inhibitory effect of the carboxylated form (Uy234-C) on *A. baumannii* AE12. Peptide concentration: 100 μM.

Once the basic physicochemical parameters for each peptide had been estimated, our next objective was to determine the bactericidal potential of each one on two pathogenic microorganisms. Hence, we evaluated Gram-positive bacteria, i.e., *S. aureus*, and Gram-negative *A. baumannii* (clinical isolate MDR). To do this, we carried out radial diffusion assays for each peptide, in triplicate. Our results, shown in Figure 2, revealed that both strains, are sensitive to the parent peptide (Uy234) but that the substitution of the proline residue at position 9 with alanine (mutant P9A) clearly inactivates this peptide, rendering it incapable of causing cell death under such conditions. On the other hand, when the C-terminal carboxyamidation of the parent peptide is modified and the typical carboxyl group is exposed; the resulting peptide (Uy234-C) was able to exert a slight inhibitory effect only against *A. baumannii* but not against S. aureus. Therefore, we can state that, to our knowledge, this is the first report that, for this multidrug-resistant clinical isolate of *A. baumannii* AE12, the Uy234 peptide can inhibit the cell growth. Furthermore, previous studies from our group demonstrated low cytotoxicity of Uy234 in hemolytic assays, indicating minimal effects on human erythrocytes (Cesa-Luna et al., 2019: Salazar-Hernández et al., 2024).

**FIGURE 3:**
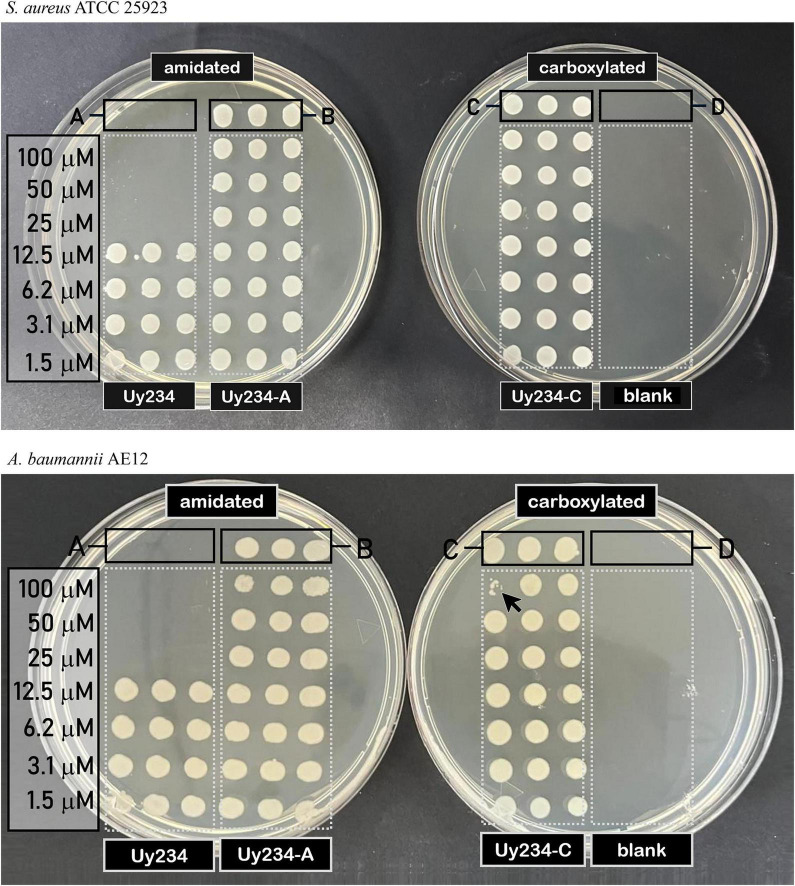
Minimum bactericidal concentration (MBC) assay of *S. aureus* ATCC 25923 and *A. baumannii* AE12 for Uy234 and its two inactivated variants. The treatments studied by radial diffusion of each peptide corresponds to the values indicated in the left panel. The parent peptide Uy234, P9A mutant (Uy234-A), and carboxylated Uy234-C, and the positive control with vancomycin is shown. The arrow indicates a slight inhibitory effect of the carboxylated form of Uy234 against *A. baumannii* at the highest concentration evaluated **(A)** Culture medium without bacteria; **(B)** with bacteria; **(C)** diluent with bacteria; **(D)** vancomycin, 50 μg/mL. Blank: acetonitrile: distilled water (1:5, v/v).

With these first experimental results, we then decided to determine the minimum inhibitory concentration (MIC) and the minimum bactericidal concentration (MBC) for each peptide studied. The results shown in Figure 3 and Table 2 confirm that the non-amidated form of Uy234, as well as the P9A mutant, are inactivated peptides against *S. aureus* ATCC 25923, while the parental peptide shows a MIC of 12.75 μM and a MBC of 25 μM. These results confirm those obtained in the radial diffusion assay and allow us to evaluate the inactivation of the modified peptides in terms of some of their physicochemical properties described above. Regarding the effect on *A. baumannii* AE12, our results indicate that, as in the previous case, the minimum dose required to achieve a bactericide effect on this Gram-negative pathogen is also 25 μM for the amide form of Uy234 (Table 2). The P9A mutant is an inactivated peptide against *A. baumanni* AE12 (Figure 3 and Table 2). However, we also note that, consistent with the radial diffusion assay shown in Figure 2, a 100 μM dose of the peptide in its carboxylated form (Uy234-C) is also capable of exerting a slight inhibitory effect on this bacterium (Figure 3). We attribute the fact that this result was obtained in only one of three replicates for this experiment to possible effects of intraspecific differential sensitivity (subpopulation heteroresistance), as has been suggested elsewhere (Tiseo et al., 2023). Thus, this slight inhibitory effect could also explain the absence of a clear inhibitory halo in solid medium, unlike that obtained with the amide form of Uy234 (Figure 2).

**TABLE 2:** Determination of the minimum inhibitory concentration (MIC) and minimum bactericidal concentration (MBC) for Uy234, and their two derivatives.

Peptide	Sequence	MIC (μM) *S. aureus*	MBC (μM) *S. aureus*	MIC (μM) *A. baumannii*	MBC (μM) *A. baumannii*
Uy234	FPFLLSLIPSAISAIKRL-NH**_2_**	12.75 ± 6.3	25	15.9 ± 0.1	25
Uy234-C	FPFLLSLIPSAISAIKRL-COOH	> 100	>100	> 100	>100
Uy234-A	FPFLLSLI**A**SAISAIKRL-NH**_2_**	> 100	>100	> 100	>100

### 3.3 Membrane damage on *S. aureus* ATCC 25923

**FIGURE 4:**
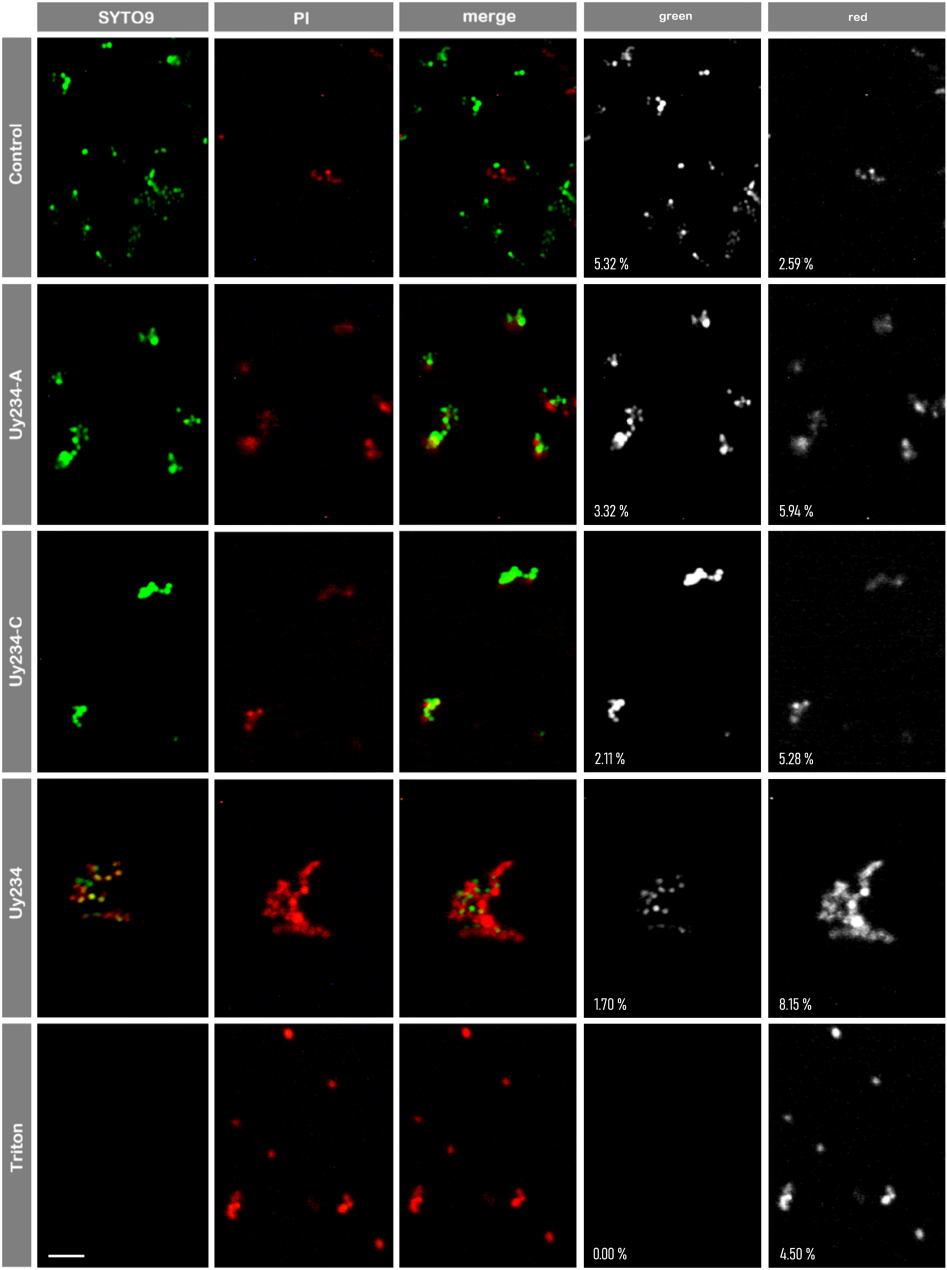
BacLight staining of live/dead *S. aureus* ATCC 25923. Cell cultures were exposed to the Uy234 peptide and its two inactivated derivatives (Uy234-C, Uy234A). The native peptide is a clearly bactericidal agent at the concentrations tested (100 μM for 2 hours of exposure). Bacteria with intact cell membranes exhibit green fluorescence (∼500 nm), whereas bacteria with damaged membranes exhibit red fluorescence (∼620 nm). Pixel analysis is presented as a percentage of green and red colors as depicted. Bar: 5 μm.

In some studies, viability in bacterial cells has been successfully monitored using the LIVE/DEAD^®^ BacLight™ stain (Pazos-Rojas et al., 2019; Robertson et al., 2019). In these experiments, dead cells are identified by red staining with propidium iodide (PI), a DNA-intercalating agent that is excluded from viable cells with intact plasma membranes. PI can penetrate the cell and bind to DNA only when the plasma membrane has lost its integrity, as occurs in membrane-compromised dead cells. Upon binding, PI intercalates into DNA with a stoichiometry of approximately one molecule per 4–5 base pairs, producing a fluorescence emission peak near 617 nm (Almheid, 1990). Cells with intact membranes, on the other hand, emit green fluorescence (498 nm/DNA, 501 nm/RNA), because this synthetic nucleic acid dye is able to penetrate the intact cell membrane of living bacteria (McGoverin et al., 2020). Here, we used this strategy to evaluate the impact of Uy234 and their two inactivated variants on *S. aureus*. These results are shown in Figure 4 and clearly indicate that the native form of the Uy234 peptide (C-amidated and with a Pro residue at position 9) is capable of lysing bacteria, releasing their DNA. On the other hand, the non-amidated form (Uy234-C) and the P9A mutant (Uy234-A) are not capable of having a significant antagonistic effect in terms of cell viability. These results are consistent with the fluorescence levels exhibited by cells not treated with peptides and in presence of the detergent Triton X-100. Taken together, these results clearly coincide with our previous viability data (Figures 2, 3) and allow us to affirm that both amidation and the presence of the Pro-9 residue are key to the bioactivity of Uy234 against *S. aureus* by the direct destabilization of their plasma membrane. Given that the MIC results for the three peptides evaluated against *A. baumannii* were similar to those observed for S. aureus, the live/dead assay was not performed in *A. baumannii*, as no substantial differences were expected. Further fluorescence-based experiments using *A. baumannii* are currently underway to complement these findings and will be reported in due course.

### 3.4 Atomic force microscopy to detect effects on lipid membranes by the Uy234 interaction and GUV permeabilization experiments

**FIGURE 5:**
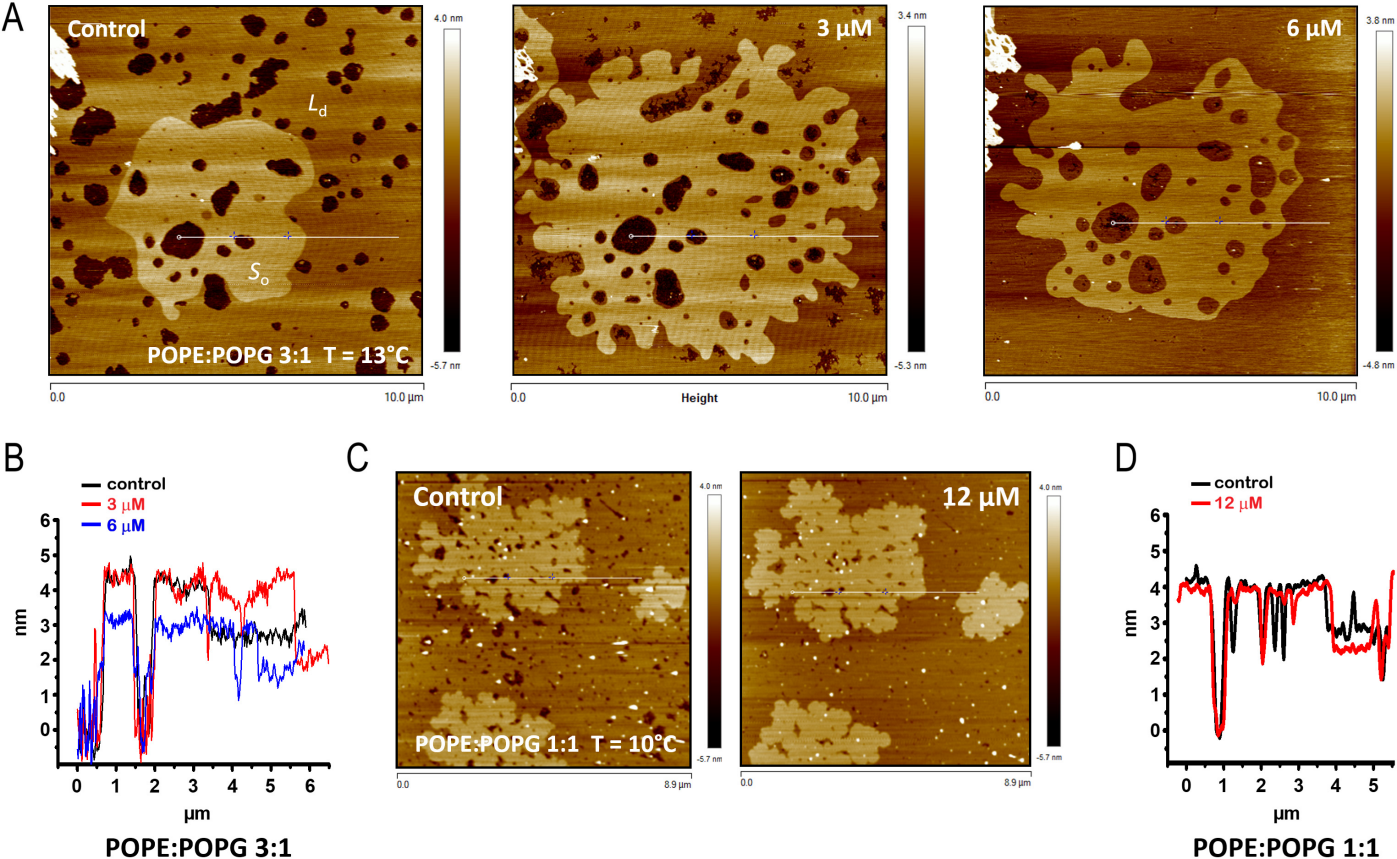
AFM images of SLB POPE/POPG exposed to Uy234. **(A)** AFM topographic image of a POPE:POPG (3:1) SLB before and after exposure to 3 and 6 μM Uy234 at 13 °C; white lines correspond to the boundaries of the substrate, the solid ordered (*S*o, rich in POPE) and liquid disordered (*L*d, rich in POPG) domains to which the sections reported in **(B)** refer to. **(B)** Average profiles of the three images in **(A)** with respect to the regions marked with a dashed white line in those images. **(C)** AFM topographic image of a POPE:POPG (1:1) SLB before (*left*) and after (*right*) exposure to 12 μM Uy234 at 10 °C. **(D)** Comparison of two-line sections corresponding to the same region of the lipid bilayer as in **(C)**.

To better understand the membrane-disrupting effects and probable membrane defects by the active form of Uy234, AFM experiments on supported lipid bilayers (SLBs) and permeability assays in GUVs were conducted. AFM imaging of SLBs is widely used with model lipid systems and provides a powerful approach to obtain nanoscale-resolution information on peptide-induced membrane structural changes (Alessandrini and Facci, 2012; Lv et al., 2018; Hammond et al., 2021). This approach has also been extensively applied to study antimicrobial peptide interactions with model membranes (Marín-Medina et al., 2018; Balleza et al., 2020; Mescola et al., 2020) Thus, we examined the impact of 3 and 6 μM of Uy234 on a POPE:POPG (3:1) bilayer supported on mica, which is a typical composition for inner cell membranes in Gram-negative bacteria (Krok et al., 2023). We concentrated on the role of the presence of different phases, solid ordered (*S***_*o*_**) and liquid disordered (*L***_*d*_**), in the lipid bilayer and, to reproduce this situation, we worked at temperatures in the range of 10-13 °C. The results depicted in Figure 5 clearly indicate a lateral expansion of the ordered lipid domains, rich in POPE in SLB when the Uy234 peptide is injected. This result suggests that Uy234, once inserted into the membrane, is capable of producing distinct effects on different membrane domains. In particular Uy234 is able to increase the area of the ordered domains (rich in POPE), probably due to the peptide entering the lipid bilayer and increasing the lateral pressure, but, at the same time, the thickness of the lipid bilayer solid phase decreases, highlighting the increase of the disorder in the bilayer. Also, the disordered regions, rich in POPG, expand, as can be confirmed by filling the lipid-free spaces in the model membrane. Moreover, this phase appears thinner in the presence of Uy234 with respect to the control condition, since the thickness of the membrane (4.5 nm) decreases to 3.1 nm as a result of exposing the bilayer to 6 μM Uy234. Notably, we did not observe any apparent thinning when the concentration of this peptide was only 3 μM. In addition, the boundary of the *S***_*o*_** domain in the presence of the peptide appears rougher, probably reflecting also an effect of the peptide on the line tension between the domains, as reported for another antimicrobial peptides (Henderson et al., 2016). In another series of experiments (Figure 5C), we attempted to increase the membrane’s surface electrostatic potential by using a POPE:POPG (1:1) system, thereby increasing the anionic charge of the bilayer. Under those conditions, we tested concentrations up to 12 μM of Uy234 and observed the same fluidizing effect of both phases of this binary system, even if this time, probably due to the relative increase of the presence of the negatively charged lipid POPG, the increase of the solid ordered phase is less with respect to the previous case and the disordering effect on both the solid and liquid phase is less evident, as highlighted by the comparison of the line sections in Figure 5D. Furthermore, the rough appearance of the edges of these membranes was even more noticiable.

**FIGURE 6:**
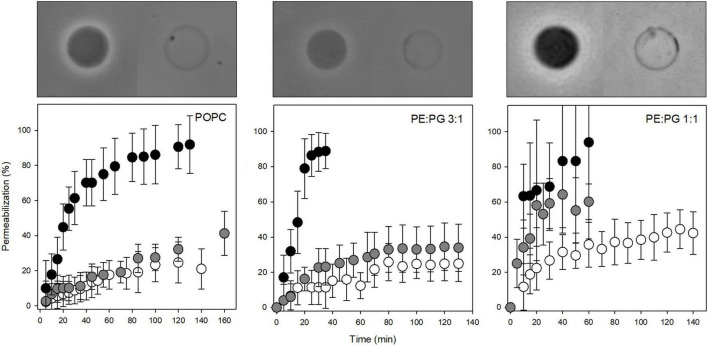
Time course of Uy234 peptide-induced membrane permeabilization in GUVs. Liposomes were formed with POPC, POPE:POPG (3:1), and POPE:POPG (1:1). Each condition was evaluated at three different peptide concentrations: 1 μM (*white*); 3 μM (*gray*); 6 μM (*black*). Upper panels show the difference in contrast of the liposomes before (*left*) and after (*right*) being permeabilized by the peptide (*n* = 3).

Next, we decided to electroform GUVs with three lipid compositions, i.e., POPC, and POPE:POPG (3:1; 1:1), and evaluate liposome permeabilization induced by Uy234 using a phase contrast microscopy permeability analysis. Due to differences in density in these experimental preparations, the vesicles tend to settle at the bottom of the BSA-passivated glass chamber under the influence of gravity. Under these conditions, and given the differences in refractive indices between glucose (outside) and sucrose (inside), suitable conditions are achieved for measuring changes in vesicle contrast and assessing potential permeability effects in the vesicles (Mescola et al., 2019). Although electroformation efficiency was lower for PE-rich lipid mixtures, our results clearly indicate that the presence of anionic lipids (PG) facilitates the permeabilization of the GUV membrane in a dose-dependent manner (Figure 6). Likewise, we corroborate that the presence of a zwitterionic lipid (PC), although it also facilitates the permeabilization of GUVs, it does not compromise their global integrity, as it can withstand high doses of that peptide (6 μM) for very long periods of time (up to 2 h), something that was not detected when the same treatment included liposomes with a global anionic charge and where, at the same dose, in 40 min (PE:PG, 3:1) or 1 h (PE:PG, 1:1), most vesicles disappeared from the field of observation within 40–60 min.

In addition to these results, we wanted to monitor in real time the permeabilizing effect of Uy234 on lipid bilayers. To do this, we formed DOPC GUVs, a lipid that allows electroforming liposomes with diameters larger than 20 μm, clearly visible under light microscopy by phase contrast. As shown in Supplementary Movie 1, these vesicles collapse upon interaction with Uy234 (1 μM). We have interpreted this as a perturbation of the integrity of the lipid bilayer in the larger vesicles (*white* arrows). Under these conditions, we propose that the Uy234 peptide induces a disruption of lipid packing and promotes lytic activity in such model membranes. In other vesicles, specifically the smallest ones (*black* arrows), we observed only the permeabilizing effect of this peptide. Next, with the aim of contrasting the inactivating effect of the P9A mutation, we qualitatively compared, using liposome microaspiration with a pipette, the effect of both peptides, i.e., Uy234 and Uy234-A, both exposed to a concentration of 1 μM of each peptide on the lipid bilayer stability. As shown in Supplementary Movies 2, 3, Uy234-A is not capable of permeabilizing or collapse the lipid membrane; in contrast, exposure of these vesicles to Uy234 destroys the GUVs practically as soon as the active peptide is injected near to the liposome. This observation was consistent in several repetitions of this assay. In those experiments, a small pressure was applied with the micropipette in order to have a lateral tension of ∼1 mN/m on the lipid bilayer (Supplementary Movies).


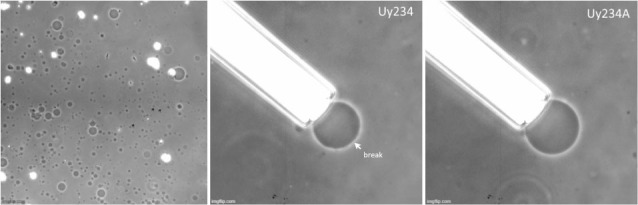


### 3.5 Molecular dynamic simulations

**FIGURE 7:**
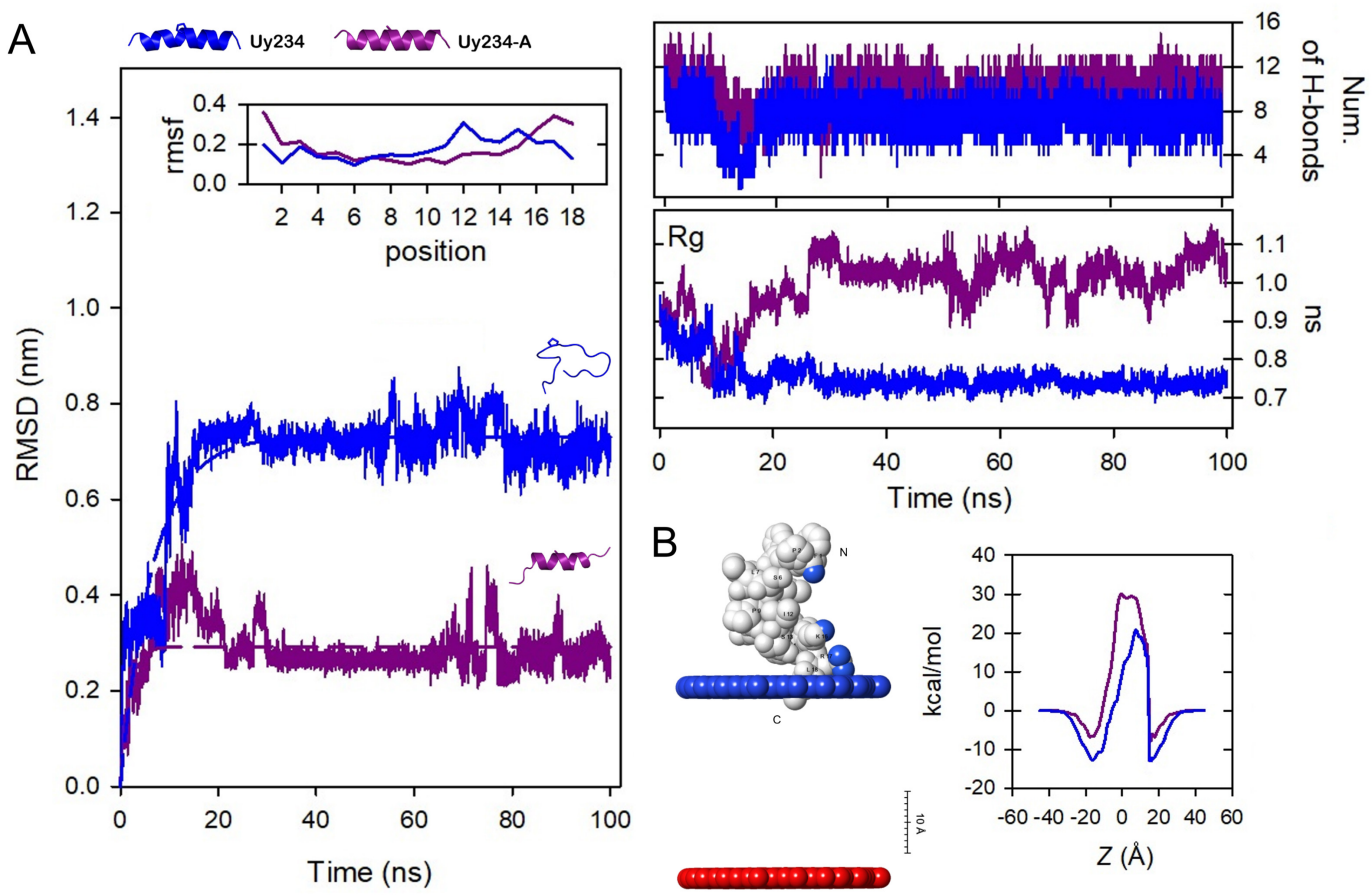
Molecular dynamic simulations of Uy234 and Uy234-A in aqueous milieu. **(A)** RMSD, RMSF, Radius of gyration (Rg), and average H-bonding comparisons for Uy234 and Uy234-A peptides in MD simulations. The substitution of the Pro9 residue with Ala keeps Uy234-A conformationally more ordered over long periods of time in aqueous phase, which is consistent with a high number of H-bonds and greater compactness of the structure. **(B)** Energy associated with the adsorption and penetration of the Uy234 (*blue*) and Uy234-A (*purple*) peptides. The blue and red pseudoatoms mark the hydrophobic boundaries of the lipid bilayer in close interaction with Uy234. The energy plot highlights the maximum energy required by each peptide to traverse a DOPC membrane at 300 K and pH 7.

Finally, given the clear differences in lytic capabilities and the limited antibacterial power shown by Uy234-A, we wondered what effect the P9A substitution would have and how it would impact the performance of this peptide in aqueous phase. Hence, we decided to compare the dynamic behavior of the two peptides (Uy234 and Uy234-A) in aqueous solution, as we were interested in clearly revealing the conformational freedom of each peptide, as we have recently reported for other peptides found in the venom of *U. yaschenkoi* (Salazar-Hernández et al., 2024; Fernández-Sánchez et al., 2025). Using the GROMACS package and the Gromos 43a1 FF, we evaluated the conformational freedom of both the wild-type peptide and the P9A mutant. Our results, shown in Figure 7A, consistently indicated (n = 3) that the Pro9 residue confers significant structural mobility to Uy234, disrupting the α-helical model peptides and reducing the formation of intramolecular H-bonds in such structures. This result is consistent with our previous proposal, in which we argued that this central proline residue is capable of conferring significant conformational freedom to Uy234 to be active. Trying to find computational evidence to support this hypothesis, we also simulated the energy associated with the penetrability of each peptide in DOPC membranes. Thus, in Figure 7B, we found a logarithmic permeability coefficient (logP) of -13.9 kcal/mol for the active peptide (Uy234) and -21.06 kcal/mol for the P9A mutant (Uy234-A). It should be noted that calculated logP values > -5 kcal/mol indicate that the peptide can easily cross the lipid bilayer (Lomize and Pogozheva, 2018). Considering this evidence and associating it with the rest of our experimental data, our results suggest that the presence of an alanine residue at this position prevents Uy234 from being internalized, which reduces the bioactivity of this mutant peptide.

## 4 Discussion

In this study, we demonstrate that the antimicrobial activity of the short peptide Uy234, derived from the Australian scorpion *Urodacus yaschenkoi*, against *S. aureus* and *A. baumannii* depends fundamentally on two structural determinants: the presence of a central Pro residue at position 9 and the C-terminal amidation. These findings highlight how subtle modifications in the sequence can profoundly alter biological function, even when some of the overall physicochemical parameters for these peptides remain virtually unchanged. However, it is noteworthy that the P9A mutant significantly increases both the rigidity and the electrostatic potential, as well as the Boman index for this peptide. This metric quantifies the protein-binding potential, which estimates how likely a peptide is to interact with other proteins/peptides (Boman, 2003). In this context, it is very notable that the propensity for aggregation *in vitro* for Uy234-A is very high (Table 1), which is also consistent with the high energy associated with its internalization into DOPC model membranes and the high degree of compactness that Uy234-A exhibits compared to the high degree of disorder characteristic of Uy234 (Figure 7).

As indicated above, our data show that the presence of the C-amide end is also critical for the bioactivity of Uy234, at least against S. aureus ATCC 25923 and A. baumannii AE12. However, to our surprise, the carboxylated form of this peptide (Uy234-C) also exhibits a slight growth-inhibitory effect against this Gram-negative pathogen (Figure 2). This data is interesting but not unusual, as a similar effect has recently been reported for Temporin-PMb secreted by *Lithobates palmipes* (Ranidae) in its carboxylated form (FLPFLGKLFSGIF-COOH) (Barbosa et al., 2025). In another study, Latarcin-3a (SWKSMAKKLKEYMEKLKQRA-COOH), a non-amidated spider-derived peptide, inhibited the growth of *A. baumannii* but not of *S. aureus* (de Moraes et al., 2022). While the mechanism of action for those peptides is still unknown, we hypothesize that membrane-lipid composition among both bacteria may be the key factor. In any case, it is also interesting that both Temporin-PMb and Latarcin-3a are peptides with a very low propensity for aggregation *in vitro*, i.e., 14.15 and 0.00, respectively. On the other hand, the intrinsic flexibility of both peptides is also significantly higher compared to the Uy234 P9A mutant, with an mBf value of 1.71 and 3.02, respectively (*data not shown*). These preliminary analyses suggest that, in addition to the lipid component of the target cells, peptides as short as Uy234 (18 aa) or Temporin-PMb (13 aa) require high atomic mobility, capable of keeping the peptides in an unfolded state while they are in the aqueous phase and poorly aggregated in consequence.

Similarly, we have evidence that the Uy234-A mutant is also harmless against *A. baumannii* AE12 (Figure 3). Even so, this substitution significantly increases both the MIC and MBC in both bacteria (Table 2). Proline is often described as a helix-disrupting residue (Yun et al., 1991; Nilsson et al., 1998); however, in short AMPs such structural discontinuities can be functionally advantageous. Proline may introduce localized flexibility or a subtle bend within the helix, facilitating membrane insertion, reorientation, or transient pore formation, as it has been described for several AMPs (Balleza, 2025). Thus, replacing proline with alanine, a strong helix stabilizer (Rohl et al., 1999), may increase helical rigidity but impair the dynamic conformational adaptability required for effective membrane destabilization. The LIVE/DEAD™ BacLight™ assay depicted in Figure 4 could indicate that the role of central proline (P9) in Uy234 is therefore crucial for killing *S. aureus* ATCC 25923 bacteria. However, we must also mention that although the MICs in the case of *A. baumannii* AE12 were similar with this peptide (Table 2), this does not necessarily imply identical mechanisms of action. Therefore, the bioactivity of Uy234 in *A. baumannii* AE12 remains to be determined using this cell viability assay. Analysis of membrane integrity also revealed that after treatment with Uy234, the use of SYTO9/PI fluorophores allow membrane rupture effects to be detected, at least in the Gram-positive model, i.e., *S. aureus*, since PI only permeates damaged or dead cells, emitting red fluorescence (Almheid, 1990). However, the pixel analysis we conducted in this experiment may have included some false positives related to the tendency of PI to bind nonspecifically (Rieger et al., 2010). Even so, the strong correlation between antimicrobial activity (Figures 2, 3) and membrane damage (Figure 4) supports a membrane-disruptive mechanism of action of Uy234. Likewise, the scarce membrane permeabilization we observed by using the inactive variants also in the case of DOPC vesicles, reinforces the proposal that both Pro9 and C-terminal amidation are necessary to achieve the structural configuration for effective lipid bilayer destabilization.

Based on this evidence, and once the role of these chemical modifications was studied, we decided to further investigate the interaction of Uy234 and its P9A mutant in synthetic liposomal systems, including SLBs and GUVs. As expected, the strong cationic character of Uy234 allows it to interact favorably with anionic membranes, which mimic the typical composition of bacteria. In this context, a noticeable expansive effect on lipid membranes was observed, along with a clear dose-dependent thinning that we interpreted as a fluidization in SLB (PE:PG, 3:1) (Figure 5). These effects have been widely described in the literature for peptides with pore-forming activity (Mecke et al., 2005; Rakowska et al., 2013; Marín-Medina et al., 2018). This evidence, together with the disruptive effect on living cells revealed by fluorescence (Figure 4), suggests that Uy234 binds electrostatically through high-affinity adsorption to the outer monolayer, generating a membrane thinning effect once the peptide in the aqueous phase transitions from highly disordered configurations to more ordered ones at the lipid/water interface. It should be noted, Uy234 does not appear to be long enough to span the complete thickness of the membrane, although we also have evidence of permeabilization and consistent with membrane permeabilization in GUVs formed with the same lipid composition and the inability of the P9A mutant to permeabilize/lyse DOPC membranes (Figure 6 and Supplementary Movies 2, 3). Thus, we hypothesize a mechanism of dimerization and possibly lipid-specific aggregation phenomena capable of forming stable pores for this small peptide. This proposal has been previously defended by some authors (Lorenzón et al., 2019; Syryamina et al., 2022).

Taken together, these data and the results of our molecular simulations (Figure 7) support a mechanism of action strongly dependent on conformational dynamics for a highly hydrophobic peptide but with such intrinsic flexibility that the presence of a key residue (P9) destabilizes helical configurations, keeping it disordered in the aqueous phase. Thus, we can propose a model of the mechanism of action of Uy234, where the high conformational freedom of Uy234, facilitated by the presence of a central proline residue and the effect of C-terminal carboxyamidation, allows it to more easily overcome the energy barrier that prevents this peptide from interacting with the lipid membrane. Furthermore, we have evidence of thinning and fluidizing effects on solid phases in model lipid systems and dose-dependent permeabilization of giant liposomes (GUVs). These data emphasize that subtle structural determinants in small AMPs govern biological activity and should be carefully considered in the rational optimization of new therapies against several bacterial pathogens.

## 5 Conclusion

In this study we have obtained convincing evidence of the key role that both C-terminal carboxyamidation and the presence of the central proline residue in the bioactivity of the peptide Uy234 against *S. aureus* ATCC 25923 and *A. baumannii* AE12. This peptide is present in the venom of the scorpion *U. yaschenkoi*. The evidence we present in this work to support this proposal derives from multiple experimental techniques, as well as from bioinformatic analysis of this peptide and its inactivated derivatives. We have also concluded that the structural parameters of Uy234, such as its intrinsic flexibility and conformational freedom, are key to its performance as antimicrobial. With this type of comprehensive study, it is feasible to consider rational optimization strategies capable of combating bacteria that have acquired multi-resistance to various conventional antibiotics.

## Author’s note

Preliminary data obtained in our laboratory indicate that the Uy234 also exhibits activity against *P. aeruginosa* ATCC 25619. These results are preliminary and are currently being validated. In any case, we have also reported the bioactivity of Uy234 against several bacterial strains (Salazar-Hernández et al., 2024).

## Data Availability

The original contributions presented in this study are included in the article/Supplementary material, further inquiries can be directed to the corresponding authors.

## Footnotes

1
https://simlab.uams.edu/

## Supplementary material

Supplementary Figure 1 Antimicrobial susceptibility of *Acinetobacter baumanii* AE12 evaluated by disk diffusion assay

Supplementary Figure 2 Ramachandran plot of the main chain phi (φ) and psi (ψ) angles for Uy234 (*left*) and Uy234A (*right*) peptides. Dots represent allowable combinations and the spaces prohibited ones of those angles. The P9A mutation has a greater effect on the structure of this peptide. *Errat* plots of both peptides are depicted. On the error axis (*), two lines are drawn to indicate the confidence with which it is possible to reject regions that exceed that error value.

Supplementary Movie 1 Permeabilizing (*black* arrows) and lytic (*white* arrows) effect of Uy234 on GUVs of DOPC.

Supplementary Movie 2 Phase contrast movie of a GUV (DOPC) exposed to peptide Uy234 (1 μM). Vesicle diameter ≈ 25 μm.

Supplementary Movie 3 Phase contrast movie of a GUV (DOPC) exposed to peptide Uy234-A (1 μM). Vesicle diameter ≈ 25 μm.
