# Supplementary figures and images for: Structural determinants of the scorpion venom peptide Uy234 govern bactericidal activity and membrane-disruptive properties
Source: Front Microbiol. 2026 Jun 11;17:1830314. doi: 10.3389/fmicb.2026.1830314 (PMC13294106; doi:10.3389/fmicb.2026.1830314)

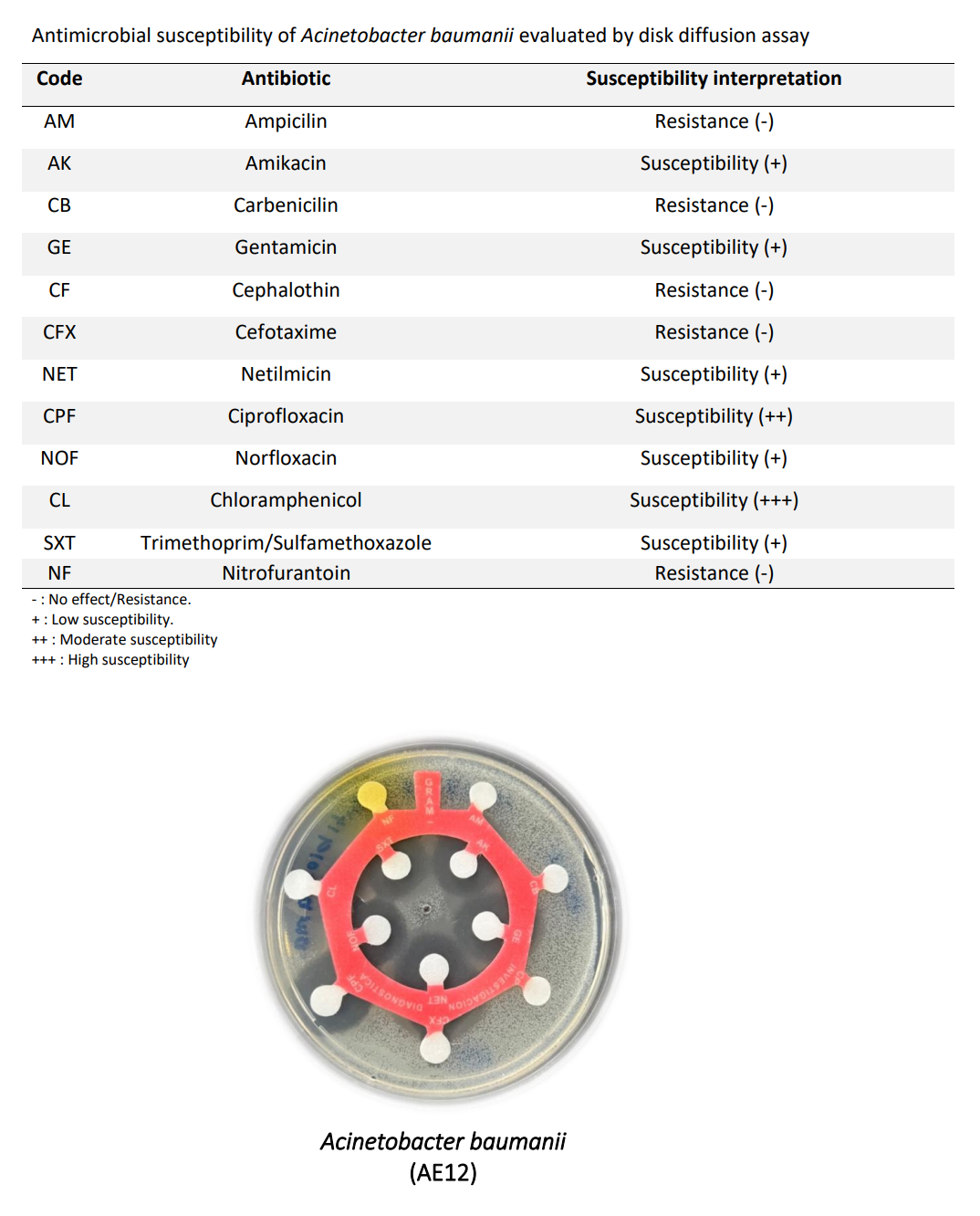

Supplement: Supplementary Figure 1 — Antimicrobial susceptibility of Acinetobacter baumanii AE12 evaluated by disk diffusion assay [file Image_1.PNG]

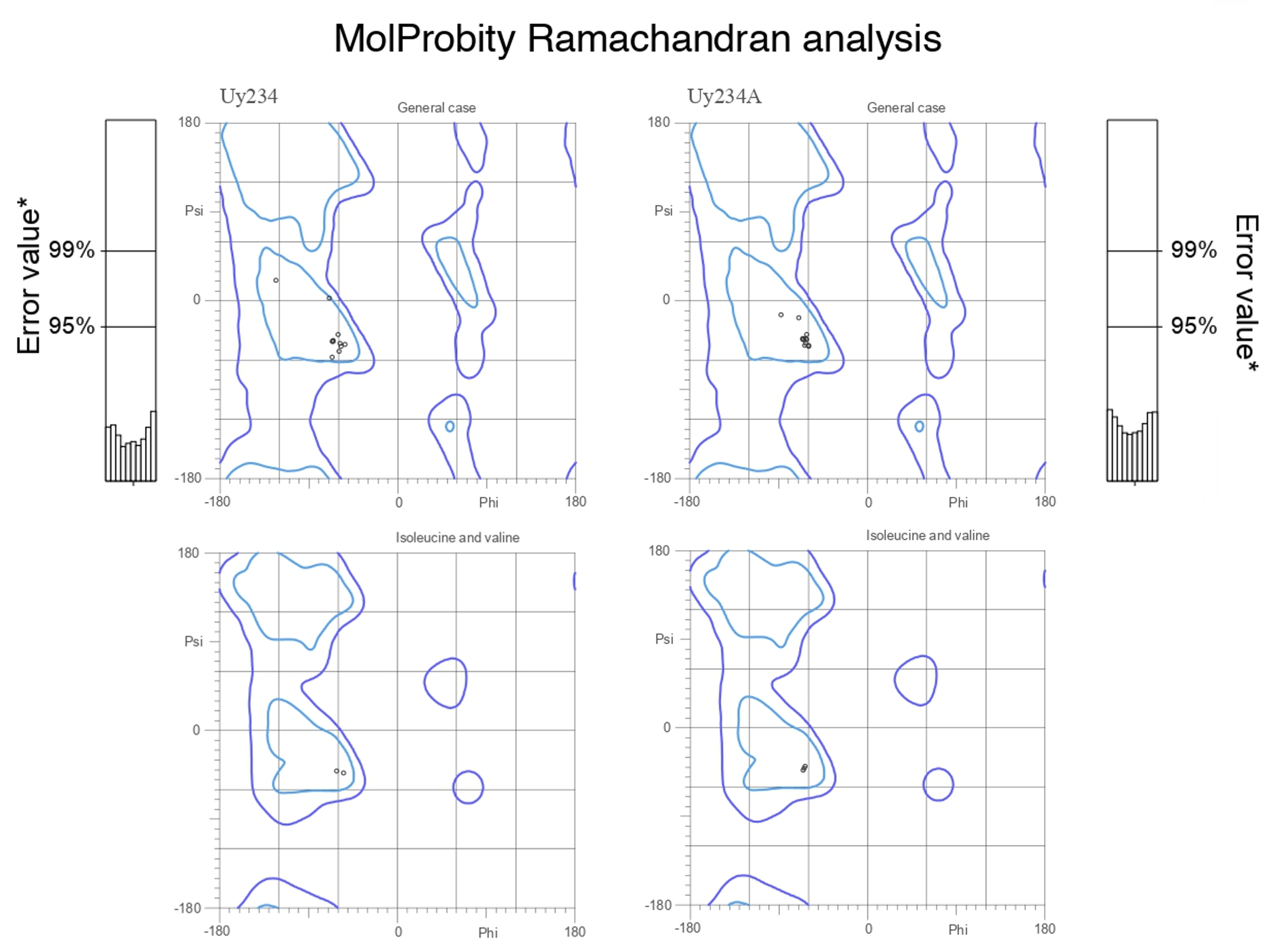

Supplement: Supplementary Figure 2 — Ramachandran plot of the main chain phi (φ) and psi (ψ) angles for Uy234 (left) and Uy234A (right) peptides. Dots represent allowable combinations and the spaces prohibited ones of those angles. The P9A mutation has a greater effect on the structure of this peptide. Errat plots of both peptides are depicted. On the error axis (*), two lines are drawn to indicate the confidence with which it is possible to reject regions that exceed that error value. [file Image_2.png]
